# 1-Benzyl-1,2,3,4-tetrahydroisoquinoline, an Endogenous Neurotoxic Compound, Disturbs the Behavioral and Biochemical Effects of l-DOPA:* In Vivo* and *Ex Vivo* Studies in the Rat

**DOI:** 10.1007/s12640-014-9476-x

**Published:** 2014-05-20

**Authors:** Agnieszka Wąsik, Irena Romańska, Jerzy Michaluk, Małgorzata Kajta, Lucyna Antkiewicz-Michaluk

**Affiliations:** 1 Department of Neurochemistry, Institute of Pharmacology Polish Academy of Sciences, 12 Smetna Street, 31-343 Kraków, Poland; 2 Department of Neuroendocrinology, Institute of Pharmacology Polish Academy of Sciences, 31-343 Kraków, Poland

**Keywords:** 1-Benzyl-1,2,3,4-tetrahydroisoquinoline, l-DOPA, Parkinson’s disease, Microdialysis study, Caspase-3 activity, Dopamine

## Abstract

Environmental factors and endogenously produced toxins, such as 1-benzyl-1,2,3,4-tetrahydroisoquinoline (1BnTIQ), are considered to be involved in the pathogenesis of Parkinson’s disease (PD). In this study, we investigated the impact of single and multiple 1BnTIQ (25 and 50 mg/kg i.p.) administration on l-DOPA-induced changes in the rate of dopamine and serotonin metabolism in the rat brain. Additionally, using* in vivo* microdialysis, we measured the impact of acute and multiple 1BnTIQ administrations on l-DOPA-induced dopamine release in the striatum. These data were compared with results from behavioral tests in which we measured the effect of 1BnTIQ and l-DOPA on locomotor activity. Finally, we determined the effect of the repeated administration of 1BnTIQ on the l-DOPA-induced elevation of caspase-3 activity in the hippocampus. An* ex vivo* neurochemical study indicated that both acute and chronic 1BnTIQ injections strongly inhibited l-DOPA-induced increases in the concentration of dopamine and all of its metabolites in dopaminergic structures. In contrast,* in vivo* microdialysis studies suggested that the differences in 1BnTIQ’s effects are dependent on the type of treatment. A single dose of 1BnTIQ intensified the elevation of dopamine release induced by l-DOPA administration (~1,300 %; *P* < 0.01), while multiple administrations of 1BnTIQ significantly enhanced the basal dopamine levels while partially diminishing the effects of l-DOPA injection (~200 %; *P* < 0.01). Additionally, we found that chronic administration of 1BnTIQ completely blocked the l-DOPA-induced increase in caspase-3 activity in the hippocampus. These findings indicate that both acute and chronic administrations of 1BnTIQ disturbs the behavioral and biochemical effects of l-DOPA in the rat. The data presented from* ex vivo* and* in vivo* studies clearly suggest that 1BnTIQ’s effects may be connected with the inhibition of DAT and/or COMT activity in the brain. Furthermore, elevated endogenous levels of 1BnTIQ may pose a serious risk in PD patients undergoing l-DOPA therapy.

## Introduction

Parkinson’s disease (PD) is an age-related movement disorder characterized by a progressive loss of dopaminergic neurons of the substantia nigra and is associated with postural and behavioral abnormalities, such as bradykinesia, tremors, and rigidity (Klockgether 2004). In spite of intensive research efforts, the cause of dopaminergic cell neurodegeneration in PD remains unknown. Some factors that may be responsible for the degeneration of dopaminergic neurons are oxidative stress, mitochondrial dysfunction, protein misfolding, apoptosis, and inflammation (Gandhi and Wood 2005; Moore et al. 2005; Sas et al. 2007). Dopaminergic brain structures are particularly sensitive to oxidative stress because the metabolism of dopamine itself leads to the generation of reactive oxygen species (ROS). ROS initiate the mitochondrial-caspase cascade, which leads to the activation of the main effector, caspase-3 (Hanrott et al. 2006; Bayir et al. 2009). Currently, l-DOPA is the best treatment available for improving the symptoms of PD. l-DOPA therapy is required by all PD patients at some stage in their illness.

The pathogenesis of PD may involve both environmental factors and endogenous toxins. 1-Benzyl-1,2,3,4-tetrahydroisoquinoline (1BnTIQ), an endogenous neurotoxin, has been proposed as a possible etiological factor of idiopathic PD (Kotake et al. 1995). The concentration of 1BnTIQ in the CSF of parkinsonian patients is three times higher than that in the CSF of neurological control subjects (Kotake et al. 1995). Chronic treatment with 1BnTIQ produces parkinsonian-like symptoms in rodents and primates (Kotake et al. 1995, 2003; Kohta et al. 2010). Evidence demonstrates that 1BnTIQ induces cell death via apoptosis and dose-dependently elevates the level of the pro-apoptotic protein Bax, while decreasing the concentration of the anti-apoptotic protein Bcl-xl. Additionally, 1BnTIQ produces an increase in the formation of the active caspase-3 protein fragments (Shavali and Ebadi 2003). 1BnTIQ, which is synthesized endogenously in the brain and/or is obtained exogenously in the diet, can be taken up by neurons via dopamine transporter (DAT) (Okada et al. 1998). As such, 1BnTIQ accumulates in dopaminergic neurons, where it is thought to exert some pathological effects leading to Parkinsonism. In previous studies, we showed that 1BnTIQ significantly affects dopamine (DA) structures, producing an increase in the rate of DA metabolism together with pronounced activation of the oxidative MAO-dependent catabolic pathway (Antkiewicz-Michaluk et al. 2001; Wąsik et al. 2009). 1BnTIQ also significantly inhibits the COMT-dependent O-methylation pathway. The depression of DA levels produced by 1BnTIQ is most pronounced in the striatum and nucleus accumbens because this effect is specific to dopaminergic neurons.

The aim of this study was to determine the consequences of acute and chronic administrations of 1BnTIQ on the effects of l-DOPA in the rat brain using behavioral and biochemical assays. We used the dose of l-DOPA which produces light stimulation with simultaneously significant biochemical changes. In neurochemical experiments, we investigated l-DOPA-induced changes in the rate of DA metabolism, as well as* in vivo* DA release in different brain structures after treatment with 1BnTIQ. The effects of 1BnTIQ on l-DOPA metabolism in the striatum and l-DOPA-induced elevation of caspase-3 activity in the hippocampus were also examined. In behavioral studies, we measured the effects of acute and chronic administrations of 1BnTIQ on l-DOPA-induced locomotor hyperactivity.

## Materials and Methods

### Animals and Treatments

All experiments were carried out in male Wistar rats with an initial body weight of 220–240 g. All animals had free access to standard laboratory food and tap water and were kept at room temperature (22 °C) under an artificial light/dark cycle (12/12 h, light on at 7:00).

1-Benzyl-1,2,3,4-tetrahydroisoquinoline (1BnTIQ) was administered at a dose of 25 or 50 mg/kg intraperitoneally (i.p.) once or chronically for 14 consecutive days. In the mixed group, l-DOPA (100 mg/kg i.p.) was administered once, 15 min after the last 1BnTIQ administration. Control rats were treated with the appropriate vehicle. Rats were killed by decapitation 2 h after last drug injections and different brain structures were dissected for analysis. The experiments were carried out between 9:00 and 16:00.

All experimental procedures were carried out in accordance with the National Institutes of Health Guide for the Care and Use of Laboratory Animals and were granted an approval from the Bioethics Commission as compliant with Polish Law. All experimental procedures were approved by the Local Bioethics Commission of the Institute of Pharmacology, Polish Academy of Sciences in Kraków.

### Drugs

1-Benzyl-1,2,3,4-tetrahydroisoquinoline (1BnTIQ hydrochloride) was synthesized (according to Cannon and Webster 1957) at the Department of Drug Chemistry of the Institute of Pharmacology, the Polish Academy of Sciences in Krakow. Purity of the compound was verified by measurement of the melting point and homogeneity was assessed on a chromatographic column. l-DOPA (Sigma-Aldrich, USA) was obtained commercially. The compounds were dissolved in a 0.9 % NaCl solution.

### Behavioral Study

#### Locomotor Activity

Locomotor activity was assessed in actometers (Opto-Varimex activity monitors; Columbus Inst., USA) linked on-line to a compatible IBM PC. Each cage (43 × 44 × 25 cm) perimeter was lined with an array of 15 × 15 photocell beams located 3 cm from the floor surface as reported previously (Filip et al. 2007). Interruptions of the photocell beams were counted as a measure of horizontal locomotor activity and were defined as the distance traveled (in cm). Horizontal locomotor activity was recorded for 60 (acute treatment group) or 90 min (14-day chronic treatment group) and analyzed using the Auto-Track Software Program (Columbus Instruments, USA). Animals were placed into the actometers for a 15-min adaptation period before drug administration and behavioral testing. Rats were given 25 or 50 mg/kg 1BnTIQ i.p. either as their only acute dose (acute treatment group) or as their last dose (14-day chronic treatment group). l-DOPA (100 mg/kg i.p.) was given acutely 15 min after 1BnTIQ administration. Each group consisted of five to six animals.

### Biochemical Studies

#### *Ex Vivo* Experiments

##### DA Metabolism and l-DOPA Metabolism

Two hours after the last 1BnTIQ injection, rats were killed by decapitation, and the substantia nigra and striatum were immediately dissected. The tissue was frozen on dry ice (−70 °C) until use in a biochemical assay. The levels of DA and its metabolites, 3,4-dihydroxyphenylacetic acid (DOPAC), 3-methoxytyramine (3-MT), and homovanillic acid (HVA), as well as the l-DOPA metabolite, 3-methoxy-DOPA (3-MDOPA) were assayed by high-performance liquid chromatography (HPLC) with electrochemical detection (Hewlett Packard 1049A). The tissue samples were weighed and homogenized in ice-cold 0.1 M perchloroacetic acid containing 0.05 mM ascorbic acid. After centrifugation (10,000×*g* for 5 min), the supernatants were filtered through RC 58 0.2-im cellulose membranes (Bioanalytical Systems, West Lafayette, IN, USA). The HP 1050 chromatograph (Hewlett-Packard, Golden, CO, USA) was equipped with C18 columns. The electrochemical cell potential was 800 mV. The mobile phase consisted of 0.05 M citrate–phosphate buffer (pH 3.5), 0.1 mM EDTA, 1 mM sodium octyl sulfonate, and 3.5 % methanol. The flow rate was maintained at 1 ml/min. DA and its metabolites were quantified by chromatograph peak height in comparison with standards run on the day of analysis. Each group consisted of five to six animals.

##### Assessment of Caspase-3 Activity

Caspase-3 activity was determined according to Nicholson et al. (1995). 1BnTIQ was administered chronically at a concentration of 25 or 50 mg/kg i.p. for 14 consecutive days. In the mixed group, l-DOPA (100 mg/kg i.p.) was given once, 15 min after last 1BnTIQ administration. Rats were decapitated 3 h after the last injection. The assessment of caspase-3 activity was performed as previously described (Kajta et al. 2009). Cell lysates were incubated at 36 °C with a colorimetric substrate, Ac-DEVD-*p*NA (*N*-acetyl-asp-glu-val-asp-*p*-nitro-anilide), which is preferentially cleaved by caspase-3. The absorbance of Ac-DEVD-*p*NA was monitored continuously over 60 min with a Multiskan Spectrum Microplate Spectrophotometer (ThermoLabsystems, Vantaa, Finland). Data were analyzed with Ascent software, normalized to the absorbance in vehicle-treated cells, and expressed as the percent of control ± SEM of three to four independent experiments. The absorbance of blanks, acting as no-enzyme controls, was subtracted from each value.

#### *In Vivo* Microdialysis

Rats were anesthetized with ketamine (75 mg/kg) and xylazine (10 mg/kg) and secured in a stereotaxic frame (Stoelting, USA). Vertical microdialysis guide cannulas (Intracerebral Guide Cannula with stylet; BAS Bioanalytical, USA) were implanted into the striatum (STR) according to the following stereotaxic coordinates: A/P +1.0, L/M +2.5, and V/D –3.5 mm from bregma and dura (G. Paxinos and C. H. Watson). 7 days after surgery, microdialysis probes were inserted into the cannulas, and the striatum was perfused with an artificial cerebrospinal fluid (aCSF) consisting of 140 mM NaCl, 2.7 mM KCl, 1.2 mM CaCl_2,_ 1 mM MgCl_2,_ 0.3 mM NaH_2_PO_4_, and 1.7 mM Na_2_HPO_4_ (pH 7.4) at a flow rate of 1.5 μl/min with a microinfusion pump (Stoelting, IL, USA). Samples were collected from freely moving rats in 20-min intervals after a 3-h wash-out period. 1BnTIQ was injected (acute or chronic: for 14 consecutive days) in a 50 mg/kg i.p. and dialysis samples were collected for 180 min. In the mixed groups, l-DOPA (100 mg/kg i.p.) was administered 40 min after the last 1BnTIQ injection. All dialysates were immediately frozen on dry ice (−70 °C) until use in a biochemical assay.

Levels of DA and its extraneuronal metabolite, 3-MT, were assayed in dialysates (20 μl) using HPLC with electrochemical detection as described above.

Chromatographic data were processed using the ChemStation computer program (Hewlett Packard, USA). DA and its metabolites were quantified by chromatograph peak height in comparison with standards run on the day of analysis. At the end of the experiment, frozen brains were examined histologically for correct probe placement. Each group consisted of six animals.

#### Calculations and Statistics

A two-way analysis of variance (ANOVA) for repeated measures was used to analyze the results of the behavioral test (locomotor activity). Differences between control and experimental groups were assessed with Duncan’s post hoc test. Data from the microdialysis study (acute 1BnTIQ treatment) were analyzed by a one-way ANOVA for repeated measures. The results from experiments evaluating chronic 1BnTIQ administration were analyzed by a two-way ANOVA for repeated measures, followed by (if significant differences arose) Duncan’s post hoc test.

The results of the biochemical experiments were analyzed by a two-way ANOVA, followed, when appropriate, by Duncan’s post hoc test. The total catabolism rate of DA was calculated from the ratio of the concentration of the final DA metabolite, HVA, to the concentration of DA and was expressed as a catabolism rate index ([HVA]/[DA] × 100), as previously described in detail (Antkiewicz-Michaluk et al. 2001). These indices were calculated using the concentrations of individual tissue samples (*n* = 6).

## Results

### Behavioral Study

#### The Effect of Acute 1BnTIQ Administration on l-DOPA-Induced Hyperactivity in Rats

**Fig. 1:**
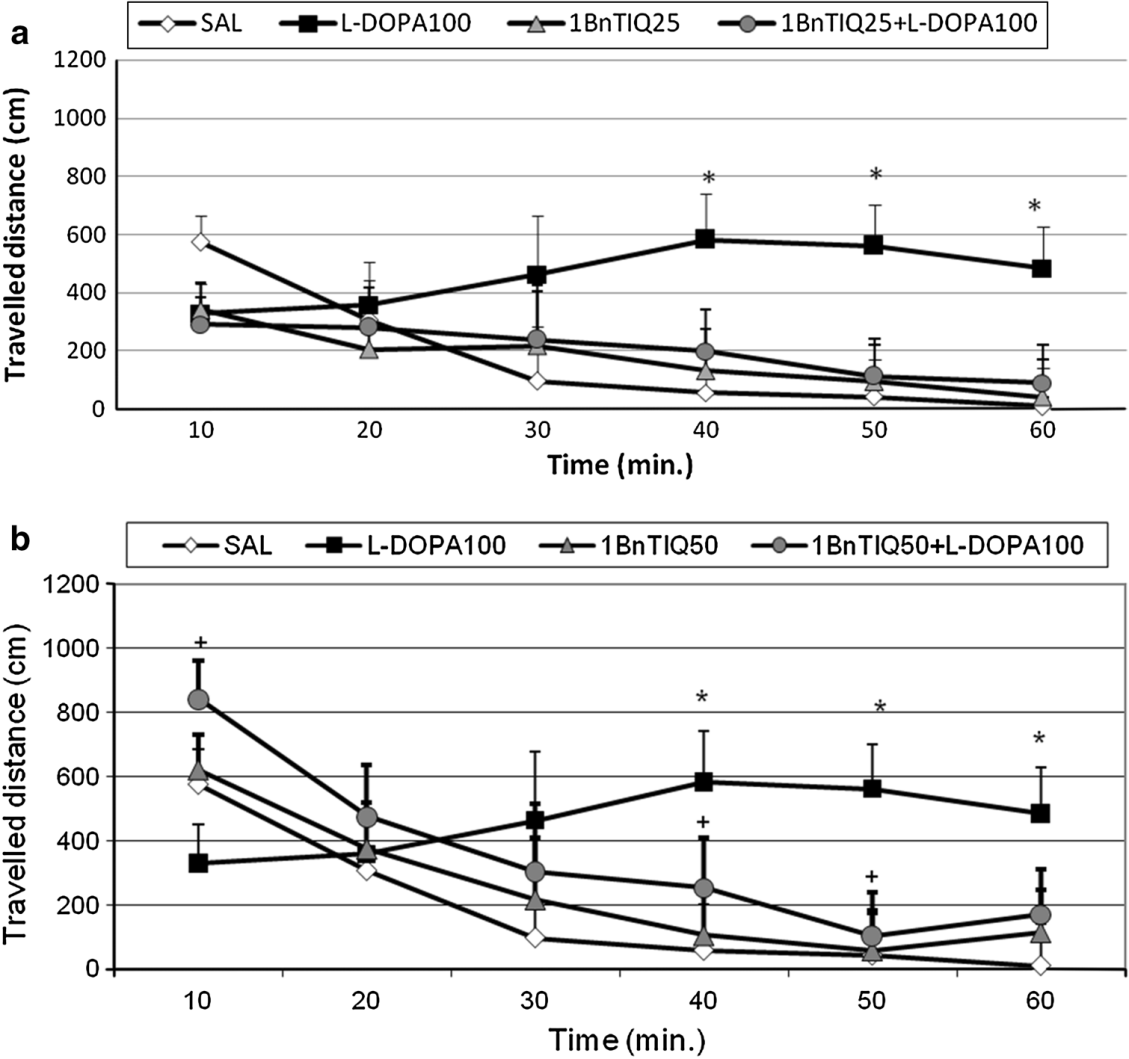
The effect of acute administration of 1BnTIQ on l-DOPA-induced hyperactivity in rats. Rats were placed into actometers and received treatments after 15 min of adaptation. Rats received a single injection of saline (control), 1BnTIQ [25 mg/kg i.p. (**a**) or 50 mg/kg i.p. (**b**)] or l-DOPA (100 mg/kg i.p.). In the mixed group, l-DOPA was injected 15 min after 1BnTIQ administration. Movements were recorded for 60 min. The data are expressed as the mean ± SEM (*n* = 5–6 animals). Data were analyzed with a two-way ANOVA for repeated measures, followed by Duncan’s post-hoc test. Statistical significance: *P < 0.05, **P < 0.01 versus saline-treated group; ^+^ *P* < 0.05 versus l-DOPA-treated group

l-DOPA (100 mg/kg i.p.) produced a significant increase in the horizontal locomotor activity of rats (*P* < 0.05) from 40 to 60 min. In the same time frame, 1BnTIQ given alone (25 mg/kg i.p.) did not change locomotor activity (Fig. 1a). In the mixed group, 1BnTIQ completely antagonized l-DOPA-induced hyperactivity.

Similarly, acute administration of 1BnTIQ at a higher dose of 50 mg/kg i.p. did not change locomotor activity, but completely antagonized l-DOPA-induced hyperactivity (Fig. 1b).

#### The Effect of Chronic 1BnTIQ Administration on l-DOPA-Induced Hyperactivity in Rats

**Fig. 2:**
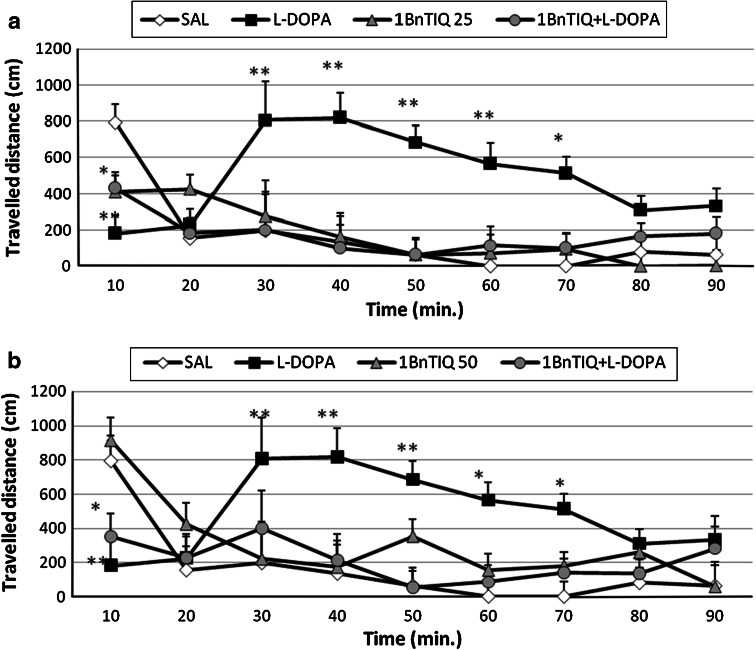
The influence of chronic administration of 1BnTIQ on l-DOPA-induced hyperactivity in rats. The influence of chronic administration of 1BnTIQ on l-DOPA-induced hyperactivity in rats. The rats were placed into actometers and after 15 min. adaptation received drugs: 1BnTIQ was administered at two doses [25 mg/kg i.p. (**a**) and 50 mg/kg i.p (**b**) chronic during 14 consecutive days]. In the mixed group, l-DOPA (100 mg/kg i.p.) was given once, 15 min after last 1BnTIQ administration. The rats received a single injection of saline (control). Next the measurement was recorded during 90 min. The data are mean ± SEM, the number of animals was *n* = 5–6; The data were analyzed by means of two-way ANOVA for repeated measures, followed when appropriate, by Duncan’s post-hoc test. Statistical significance: *P < 0.05, **P < 0.01 versus control group; ^+^ *P* < 0.05 versus l-DOPA—treated group

The chronic (14-day) administration of 1BnTIQ at a low dose of 25 mg/kg i.p. reduced (*P* < 0.05) locomotor activity only during the first 10 min of measurement (Fig. 2a). l-DOPA (100 mg/kg i.p.) produced a significant increase in horizontal locomotor activity (*P* < 0.01) from 30 to 70 min. In the mixed group, 1BnTIQ completely antagonized l-DOPA-induced hyperactivity.

Chronic 1BnTIQ administration at a higher dose of 50 mg/kg i.p. did not alter locomotor activity. The increase in locomotor activity observed with l-DOPA administration (100 mg/kg i.p.) was completely antagonized by co-administration of 50 mg/kg 1BnTIQ following chronic treatment (Fig. 2b).

### Biochemical Studies:* Ex Vivo* Experiments

#### The Effect of Acute 1BnTIQ Administration on l-DOPA-Induced Changes on DA Metabolism in Rat Brain Structures

##### Substantia Nigra

A two-way ANOVA indicated a significant effect of acute treatment with 1BnTIQ (*F*[2,29] = 10.8, *P* < 0.01) and l-DOPA (*F*[2,29] = 10.7, *P* < 0.01) on the DA concentration in the substantia nigra (Table 1). An interaction between 1BnTIQ and l-DOPA treatment was also significant (*F*[2,29] = 8.55, *P* < 0.01). Duncan’s post hoc analysis demonstrated that administration of l-DOPA strongly increased the level of DA (approximately 800 %; *P* < 0.01). In contrast, both concentrations of 1BnTIQ produced reductions in DA concentration. 1BnTIQ co-administered with l-DOPA completely antagonized l-DOPA-induced increases in DA (the DA level remained just below the control level) (Table 1).

**Table 1:** The effects of acute administration of 1BnTIQ on l-DOPA-induced changes in DA metabolism in rat brain structures

Treatment acute	Treatment acute	DA (ng/g tissue)	DOPAC (ng/g tissue)	3-MT (ng/g tissue)	HVA (ng/g tissue)	HVA/DA	
Substantia Nigra							
Saline	Saline	938 ± 100	216 ± 17	45 ± 4	111 ± 10	12 ± 1	
Saline	l-DOPA 100	7438 ± 2071**	20649 ± 5208**	162 ± 25**	4701 ± 695**	93 ± 36*	
1BnTIQ 25	Saline	534 ± 67^++^	147 ± 10^++^	24 ± 4^++^	91 ± 5^++^	18 ± 2	
1BnTIQ 50	Saline	566 ± 47^++^	171 ± 27^++^	27 ± 4^++^	101 ± 15^++^	18 ± 2	
1BnTIQ 25	l-DOPA 100	869 ± 108^++^	873 ± 273^++^	54 ± 13^++^	755 ± 300^++^	88 ± 33	
1BnTIQ 50	l-DOPA 100	774 ± 93^++^	1013 ± 493^++^	44 ± 6^++^	935 ± 466^++^	108 ± 40*	
Effect of 1BnTIQ		*F*_(2/29)_ = 10.8 *P* < 0.01	*F*_(2/29)_ = 13.5 *P* < 0.01	*F*_(2/29)_ = 19.3 *P* < 0.01	*F*_(2/29)_ = 18.7 *P* < 0.01	*F*_(2/29)_ = 0.1 N.S.	
Effect of l-DOPA		*F*_(2/29)_ = 10.7 *P* < 0.01	*F*_(2/29)_ = 16.5 *P* < 0.01	*F*_(2/29)_ = 28.9 *P* < 0.01	*F*_(2/29)_ = 45.6 *P* < 0.01	*F*_(2/29)_ = 14.8 *P* < 0.01	
Interaction of 1BnTIQ + l-DOPA		*F*_(2/29)_ = 8.55 *P* < 0.01	*F*_(2/29)_ = 13.4 *P* < 0.01	*F*_(2/29)_ = 10.1 *P* < 0.01	*F*_(2/29)_ = 18.4 *P* < 0.01	*F*_(2/29)_ = 0.1 N.S.	
Striatum							
Saline	Saline	8691 ± 265	1098 ± 43	380 ± 7	713 ± 26	8.2 ± 0.3	
Saline	l-DOPA 100	25191 ± 3967**	23040 ± 6005**	281 ± 26**	6238 ± 944**	28.1 ± 7.9*	
1BnTIQ 25	Saline	6976 ± 389^++^	1428 ± 132^++^	348 ± 25^+^	1065 ± 176^++^	15.3 ± 2.4	
1BnTIQ 50	Saline	5246 ± 411^++^	1187 ± 86^++^	271 ± 8**	907 ± 137^++^	18.2 ± 3.7	
1BnTIQ 25	l-DOPA 100	6669 ± 389^++^	2021 ± 373^++^	284 ± 12**	1865 ± 414^++^	28 ± 5.6*	
1BnTIQ 50	l-DOPA 100	5945 ± 646^++^	2330 ± 641^++^	252 ± 17**	2469 ± 755*^++^	41.8 ± 10.5**	
Effect of 1BnTIQ		*F*_(2/29)_ = 26.7 *P* < 0.01	*F*_(2/29)_ = 11.1 *P* < 0.01	*F*_(2/29)_ = 8.5 *P* < 0.01	*F*_(2/29)_ = 8.5 *P* < 0.01	*F*_(2/29)_ = 2.0 N.S.	
Effect of l-DOPA		*F*_(2/29)_ = 16.0 *P* < 0.01	*F*_(2/29)_ = 14.3 *P* < 0.01	*F*_(2/29)_ = 17.4 *P* < 0.01	*F*_(2/29)_ = 35.8 *P* < 0.01	*F*_(2/29)_ = 13.8 *P* < 0.01	
Interaction of 1BnTIQ + l-DOPA		*F*_(2/29)_ = 15.2 *P* < 0.01	*F*_(2/29)_ = 11.5 *P* < 0.01	*F*_(2/29)_ = 2.5 N.S.	*F*_(2/29)_ = 11.2 *P* < 0.01	*F*_(2/29)_ = 0.4 N.S.	

1BnTIQ was acutely administered at two concentrations (25 and 50 mg/kg i.p.). In the mixed group, l-DOPA (100 mg/kg i.p.) was given once, 15 min after 1BnTIQ administration. Rats were decapitated 2 h after the injections. The results are expressed as the mean ± SEM (*n* = 5–6 animals per group). Data were analyzed with a two-way ANOVA followed by Duncan’s post hoc test. Statistical significance: * P < 0.05, ** *P* < 0.01 versus saline-treated group; ^+^ *P* < 0.05, ^++^ *P* < 0.01 versus l-DOPA-treated group

A two-way ANOVA demonstrated a significant effect of 1BnTIQ (*F*[2,29] = 13.5, *P* < 0.01), as well as l-DOPA (*F*[2,29] = 16.5, *P* < 0.01) on the concentration of DOPAC in the substantia nigra (Table 1). An interaction between 1BnTIQ and l-DOPA was also significant (*F*[2,29] = 13.4, *P* < 0.01). The post hoc analysis indicated that acute administration of l-DOPA produced massive elevation of DOPAC concentration (approximately 10,000 %; *P* < 0.01), and this effect was strongly blocked by both concentrations of 1BnTIQ (Table 1).

The statistical analysis showed a significant effect of 1BnTIQ (*F*[2,29] = 19.3, *P* < 0.01) and l-DOPA (*F*[2,29] = 28.9, *P* < 0.01) treatment on the levels of 3-MT, as well as an interaction between 1BnTIQ and l-DOPA (*F*[2,29] = 10.1, *P* < 0.01) (Table 1). Duncan’s post hoc test showed that l-DOPA induced an increase in the concentration of 3-MT (approximately 300 %, *P* < 0.01); 1BnTIQ completely antagonized this effect (Table 1).

Treatment with 1BnTIQ (*F*[2,29] = 18.7, *P* < 0.01) and l-DOPA (F[2,29] = 45.6, *P* < 0.01) had a significant effect on the HVA concentration in the substantia nigra (Table 1). This analysis also revealed a significant interaction between 1BnTIQ and l-DOPA (*F*[2,29] = 18.4, *P* < 0.01). The post hoc analysis showed that l-DOPA administration strongly increased the level of HVA (approx. 4,000 %, *P* < 0.01), and this effect was weakened by both concentrations of 1BnTIQ (Table 1).

l-DOPA (*F*[2,29] = 14.8, *P* < 0.01) also had a significant effect on the rate of DA metabolism, measured as [HAV]/[DA] (Table 1). The statistical analysis revealed no effect of 1BnTIQ on the rate of DA metabolism (*F*[2,29] = 0.1, N.S.) or interaction between 1BnTIQ and l-DOPA (*F*[2,29] = 0.1, N.S.). Duncan’s post hoc analysis demonstrated that acute doses of l-DOPA strongly increased the rate of DA metabolism (approximately 800 %; *P* < 0.01), and a similar effect was observed when 1BnTIQ (50 mg/kg) was given in combination with l-DOPA.

#### Striatum

In the striatum, a two-way ANOVA indicated a significant effect of both 1BnTIQ (*F*[2,29] = 26.7, *P* < 0.01) and l-DOPA (*F*[2,29] = 16.0, *P* < 0.01) treatments on the DA concentration (Table 1). An interaction between 1BnTIQ and l-DOPA was also revealed by the analysis (*F*[2,29] = 15.2, *P* < 0.01). Duncan’s post hoc test showed that acute dose of l-DOPA increased the DA concentration (approx. 300 %, *P* < 0.01). Both concentrations of 1BnTIQ completely antagonized this effect (Table 1).

The statistical analysis showed a significant effect of 1BnTIQ (*F*[2,29] = 11.1, *P* < 0.01) and l-DOPA (*F*[2,29] = 14.3, *P* < 0.01) treatments on DOPAC levels, as well as an interaction between 1BnTIQ and l-DOPA (*F*[2,29] = 11.5, *P* < 0.01) (Table 1). The post hoc analysis demonstrated that treatment with l-DOPA induced a massive increase in the level of DOPAC (approximately 2,000 %). This l-DOPA-induced effect was completely blocked by 1BnTIQ administration (Table 1).

A two-way ANOVA further revealed a significant effect of acute administration of 1BnTIQ (*F*[2,29] = 8.5, *P* < 0.01) or l-DOPA (*F*[2,29] = 17.4, *P* < 0.01) on 3-MT concentration in the striatum (Table 1). However, no interaction between 1BnTIQ and l-DOPA was detected in this case (*F*[2,29] = 2.5, N.S.). Duncan’s post hoc test showed that an acute dose of l-DOPA or 1BnTIQ (50 mg/kg) reduced the levels of 3-MT (approximately 30 %). A similar effect was observed in the mixed groups.

The statistical analysis indicated a significant effect of 1BnTIQ (*F*[2,29] = 8.5, *P* < 0.01) and l-DOPA (*F*[2,29] = 35.8, *P* < 0.01) treatments on HVA levels, as well as an interaction between 1BnTIQ and l-DOPA (*F*[2,29] = 11.2, *P* < 0.01). The post hoc analysis showed that treatment with l-DOPA induced a strong elevation in the level of HVA (approximately 900 %). This effect was partially antagonized by 1BnTIQ administration (Table 1).

l-DOPA also had a significant effect (*F*[2,29] = 13.8, *P* < 0.01) on the rate of DA metabolism measured as [HAV]/[DA] (Table 1). The statistical analysis revealed no effect of 1BnTIQ on the rate of DA metabolism (*F*[2,29] = 2.0, N.S.) or interaction between 1BnTIQ and l-DOPA (*F*[2,29] = 0.4, N.S.). Duncan’s post hoc analysis demonstrated that acute administration of l-DOPA increased the rate of DA metabolism (approximately 350 %; *P* < 0.05); the strongest effect was observed when 1BnTIQ (50 mg/kg) was administered concomitantly with l-DOPA (approximately 500 %) (Table 1).

*The effect of chronic administration of 1BnTIQ on* l-DOPA-*induced changes on* DA *metabolism in rat brain structures.*

#### Substantia Nigra

In the substantia nigra, a two-way ANOVA revealed no effect of chronic treatment with 1BnTIQ (*F*[2,30] = 2.08, N.S.) on the DA concentration (Table 2). The statistical analysis indicated a significant effect of treatment with l-DOPA (*F*[2,30] = 12.64, *P* < 0.01) on the level of DA. There was no significant interaction between the chronic administration of 1BnTIQ and the acute dose of l-DOPA (*F*[2,30] = 2.00, N.S.). Duncan’s post hoc test showed that the acute administration of l-DOPA produced an increase in the DA concentration (approximately 1,000 %; *P* < 0.01). Additionally, when 1BnTIQ (50 mg/kg) given chronically was coupled with an acute dose of l-DOPA, a significant increase in the level of DA was observed (approx. 700 %; *P* < 0.05) (Table 2).

**Table 2:** The effects of chronic administration of 1BnTIQ on l-DOPA-induced changes in DA metabolism in rat brain structures

Treatment chronic	Treatment acute		DA (ng/g tissue)	DOPAC (ng/g tissue)	3-MT (ng/g tissue)	HVA (ng/g tissue)	HVA/DA	
Substantia nigra								
Saline	Saline		564 ± 44	206 ± 39	37 ± 2	84 ± 9	15 ± 2	
Saline	l-DOPA 100		5556 ± 1015**	25178 ± 5309**	181 ± 24**	6410 ± 641**	129 ± 16**	
1BnTIQ 25	Saline		502 ± 23^++^	174 ± 11^++^	40 ± 3^++^	83 ± 7^++^	17 ± 2	
1BnTIQ 50	Saline		413 ± 16^++^	180 ± 16^++^	32 ± 2^++^	99 ± 8^++^	24 ± 2	
1BnTIQ 25	l-DOPA 100		1272 ± 180^+^	2210 ± 628^++^	89 ± 11^++^	1864 ± 514^++^	128 ± 30**	
1BnTIQ 50	l-DOPA 100		3987 ± 2414*	8142 ± 4355^++^	142 ± 45**	4080 ± 1394**^+^	181 ± 30**	
Effect of 1BnTIQ			*F*_(2/30)_ = 2.08 N.S.	*F*_(2/30)_ = 8.99 *P* < 0.01	*F*_(2/30)_ = 2.22 N.S.	*F*_(2/30)_ = 5.92 *P* < 0.01	*F*_(2/30)_ = 0.1 N.S.	
Effect of l-DOPA			*F*_(2/30)_ = 12.64 *P* < 0.01	*F*_(2/30)_ = 25.72 *P* < 0.01	*F*_(2/30)_ = 34.24 *P* < 0.01	*F*_(2/30)_ = 55.78 *P* < 0.01	*F*_(2/30)_ = 14.8 *P* < 0.01	
Interaction of 1BnTIQ + l-DOPA			*F*_(2/30)_ = 2.00 N.S.	*F*_(2/30)_ = 8.94 *P* < 0.01	*F*_(2/30)_ = 2.55 N.S.	*F*_(2/30)_ = 5.92 *P* < 0.01	*F*_(2/30)_ = 0.1 N.S.	
Striatum								
Saline	Saline	9793 ± 426		1486 ± 122	383 ± 16	701 ± 34	7.2 ± 0.3	
Saline	l-DOPA 100	25128 ± 2755**		33727 ± 6080**	269 ± 16**	9696 ± 870**	39.5 ± 2.4**	
1BnTIQ 25	Saline	7645 ± 525^++^		1142 ± 84^++^	410 ± 21^++^	735 ± 57^++^	9.9 ± 1.1^++^	
1BnTIQ 50	Saline	6042 ± 348^+^		1415 ± 147^++^	335 ± 14^++^	1111 ± 104^++^	18.6 ± 1.8**^++^	
1BnTIQ 25	l-DOPA 100	14653 ± 3192^++^		4283 ± 922^++^	330 ± 15*^+^	3634 ± 769*^++^	25.1 ± 4.6**^++^	
1BnTIQ 50	l-DOPA 100	18281 ± 5335**		3318 ± 550^++^	338 ± 13^++^	7452 ± 1903**	42.6 ± 2.3**	
Effect of 1BnTIQ		*F*_(2/30)_ = 2.94 N.S.		*F*_(2/30)_ = 23.8 *P* < 0.01	*F*_(2/30)_ = 4.18 *P* < 0.05	*F*_(2/30)_ = 5.75 *P* < 0.01	*F*_(2/30)_ = 14.0 *P* < 0.01	
Effect of l-DOPA		*F*_(2/30)_ = 25.54 *P* < 0.01		*F*_(2/30)_ = 36.4 *P* < 0.01	*F*_(2/30)_ = 23.67 *P* < 0.01	*F*_(2/30)_ = 66.71 *P* < 0.01	*F*_(2/30)_ = 138 *P* < 0.01	
Interaction of 1BnTIQ + l-DOPA		*F*_(2/30)_ = 1.13 N.S.		*F*_(2/30)_ = 23.2 *P* < 0.01	*F*_(2/30)_ = 7.16 *P* < 0.01	*F*_(2/30)_ = 5.62 *P* < 0.01	F_(2/30)_ = 5.9 *P* < 0.01	

1BnTIQ was chronic administered at two concentrations (25 and 50 mg/kg i.p.) during 14 consecutive days. In the mixed group, l-DOPA (100 mg/kg i.p.) was given once, 15 min after last 1BnTIQ administration. Rats were decapitated 2 h after the injections. The results are expressed as the mean ± SEM of six samples (*n* = 6 animals per group). Data were analyzed with a two-way ANOVA followed by Duncan’s post hoc test. Statistical significance: * P < 0.05, ** *P* < 0.01 versus saline group; ^+^ *P* < 0.05, ^++^ *P* < 0.01 versus l-DOPA group

A two-way ANOVA demonstrated a significant effect of chronic treatment with 1BnTIQ (*F*[2,30] = 8.99, *P* < 0.01), as well as acute l-DOPA administration (*F*[2,30] = 25.72, *P* < 0.01) on the DOPAC concentration in the substantia nigra (Table 2). An interaction between 1BnTIQ and l-DOPA was also detected (*F*[2,30] = 8.94, *P* < 0.01). The post hoc analysis showed that l-DOPA produced a massive increase in the DOPAC concentration (approximately 10,000 %; *P* < 0.01); this effect was partially blocked by chronic treatment with 1BnTIQ (Table 2).

The statistical analysis revealed no effect of chronic treatment with 1BnTIQ (*F*[2,30] = 2.22, N.S.) on the level of 3-MT (Table 2). Treatment with l-DOPA (*F*[2,30] = 34.2, *P* < 0.01) significantly affected the 3-MT concentration. There was no interaction between chronic administration of 1BnTIQ and acute administration of l-DOPA (*F*[2,30] = 2.55, N.S.). Duncan’s post hoc test showed that acute doses of l-DOPA produced an increase in 3-MT concentration (approximately 500 %; *P* < 0.01). When 1BnTIQ (50 mg/kg) given chronically was coupled with an acute dose of l-DOPA, a significant elevation in the levels of 3-MT (approx. 400 %; *P* < 0.01) was also observed (Table 2).

A two-way ANOVA demonstrated a significant effect of chronic treatment with 1BnTIQ (*F*[2,30] = 5.92, *P* < 0.01), as well as acute l-DOPA administration (*F*[2,30] = 55.78, *P* < 0.01) on the HVA concentration in the substantia nigra (Table 2). An interaction between 1BnTIQ and l-DOPA was also detected (*F*[2,30] = 5.92, *P* < 0.01). The post hoc analysis showed that l-DOPA produced a massive increase in the HVA concentration (approximately 8,000 %; *P* < 0.01); this effect was partially blocked by chronic treatment with both concentrations of 1BnTIQ (Table 2).

l-DOPA significantly (*F*[2,30] = 14.8, *P* < 0.01) affected the rate of DA metabolism measured as [HVA]/[DA] (Table 2). The statistical analysis revealed no effect of chronic administration of 1BnTIQ on the rate of DA metabolism (*F*[2,30] = 0.1, N.S.) or interaction between 1BnTIQ and l-DOPA (*F*[2,30] = 0.1, N.S.). Duncan’s post hoc analysis demonstrated that acute treatment with l-DOPA strongly increased the rate of DA metabolism (approximately 800 %; *P* < 0.01); a similar effect was observed in the mixed groups when 1BnTIQ (25 and 50 mg/kg) was coupled with acute l-DOPA (Table 2).

#### Striatum

In the striatum, a two-way ANOVA revealed no effect of chronic treatment with 1BnTIQ (*F*[2,30] = 2.94, N.S.) on the DA concentration (Table 2). In contrast, the acute l-DOPA treatment produced a significant effect on the level of DA (*F*[2,30] = 25.54, *P* < 0.01). The statistical analysis revealed no interaction between chronic administration of 1BnTIQ and l-DOPA administration (*F*[2,30] = 1.13, N.S.). The post hoc test indicated that acute doses of l-DOPA increased the level of DA (approximately 300 %, *P* < 0.01) and that this effect was antagonized only by chronic treatment with a lower (25 mg/kg) concentration of 1BnTIQ (Table 2).

A two-way ANOVA demonstrated a significant effect of chronic treatment with 1BnTIQ (*F*[2,30] = 23.8, *P* < 0.01) as well as the acute administration of l-DOPA (*F*[2,30] = 36.4, *P* < 0.01) on the DOPAC concentration in rat striatum (Table 2). An interaction between 1BnTIQ and l-DOPA was also detected (*F*[2,30] = 23.2, *P* < 0.01). The post hoc analysis showed that l-DOPA produced a massive increase in DOPAC concentration (approximately 2,000 %; *P* < 0.01); this effect was completely blocked by chronic treatment with both concentrations of 1BnTIQ (Table 2).

Chronic administration of 1BnTIQ (*F*[2,30] = 4.18, *P* < 0.05) and acute administration of l-DOPA (*F*[2,30] = 23.67, *P* < 0.01) both significantly affected the 3-MT concentration in striatum (Table 2). The statistical analysis also revealed an interaction between 1BnTIQ and l-DOPA (*F*[2,30] = 7.16, *P* < 0.01). Duncan’s post hoc test showed that l-DOPA decreased the level of 3-MT (approximately 30 %; *P* < 0.01) and this effect was reversed by chronic treatment with both concentrations of 1BnTIQ (Table 2).

A two-way ANOVA revealed a significant effect of both the chronic 1BnTIQ treatment (*F*[2,30] = 5.75, *P* < 0.01) and acute l-DOPA administration (*F*[2,30] = 66.71, *P* < 0.01) on the HVA concentration in the striatum (Table 2). An interaction between the chronic administration of 1BnTIQ and l-DOPA was also detected (*F*[2,30] = 5.62, *P* < 0.01). The post hoc analysis demonstrated that l-DOPA strongly increased the HVA concentration (approximately 1,400 %; *P* < 0.01); this effect was partially antagonized only by chronic treatment with a lower (25 mg/kg) concentration of 1BnTIQ (Table 2).

Chronic administration with 1BnTIQ (*F*[2,30] = 14.0, *P* < 0.01) and acute l-DOPA administration (*F*[2,30] = 138, *P* < 0.01) produced significant effects on the rate of DA metabolism measured as [HAV]/[DA] (Table 2). An interaction between 1BnTIQ and l-DOPA was also significant (*F*[2,30] = 5.9, *P* < 0.01). Duncan’s post hoc analysis demonstrated that chronic treatment with a higher (50 mg/kg) concentration of 1BnTIQ and acute administration of l-DOPA induced an increase the rate of DA metabolism (approximately 300 and 600 %, respectively). Only a lower dose (25 mg/kg) of 1BnTIQ given chronically could partially antagonize this effect (Table 2).

#### The Effect of Acute and Chronic Administration of 1BnTIQ on l-DOPA Metabolism in Rat Striatum

A two-way ANOVA demonstrated a significant effect of acute treatment with 1BnTIQ (*F*[1,18] = 7.38, *P* < 0.05) or l-DOPA (*F*[1,18] = 26.7, *P* < 0.01) on the 3-MDOPA concentration in striatum (Table 3). An interaction between 1BnTIQ and l-DOPA was also detected (*F*[1,18] = 7.38, *P* < 0.05). Duncan’s post hoc test indicated that l-DOPA produced a massive increase the level of 3-MDOPA (by approx. 30,000 times; *P* < 0.01); this effect was strongly blocked (by approximately 70 %; *P* < 0.01) by acute treatment with 1BnTIQ (50 mg/kg i.p.). Similarly, a two-way ANOVA revealed a significant effect of chronic administration of 1BnTIQ (*F*[2,30] = 6.18, *P* < 0.01) and acute administration of l-DOPA (*F*[2,30] = 69.8, *P* < 0.01) on the concentration of 3-MDOPA in the striatum. Here again, the interaction between 1BnTIQ and l-DOPA was significant (*F*[2,30] = 6.19, *P* < 0.01). The post hoc analysis demonstrated that l-DOPA strongly increased the 3-MDOPA concentration (*P* < 0.01); this effect was strongly inhibited by chronic treatment with a lower (25 mg/kg) concentration of 1BnTIQ (Table 3), while the higher concentration of 1BnTIQ (50 mg/kg) antagonized this effect only partially (*P* < 0.05) (Table 3).

**Table 3:** The impact of acute and chronic administration of 1BnTIQ on l-DOPA-induced increase the concentration of 3-MDOPA in rat striatum

Treatment acute	Treatment acute	3-MDOPA (ng/g tissue)
Saline	Saline	1.5 ± 0.19
Saline	l-DOPA 100	48687 ± 8801**
1BnTIQ 50	Saline	1.43 ± 0.17
1BnTIQ 50	l-DOPA 100	15125 ± 3468^++^
Effect of 1BnTIQ		*F*_(1/18)_ = 7.38 *P* < 0.05
Effect of l-DOPA		*F*_(1/18)_ = 26.7 *P* < 0.01
Interaction of 1BnTIQ + l-DOPA		*F*_(1/18)_ = 7.38 *P* < 0.05

Treatment chronic	Treatment acute	3-MDOPA (ng/g tissue)
Saline	Saline	1.49 ± 0.22
Saline	l-DOPA 100	26868 ± 3867**
1BnTIQ 25	Saline	1.52 ± 0.24
1BnTIQ 50	Saline	1.17 ± 0.12
1BnTIQ 25	l-DOPA 100	8381 ± 1714^++^
1BnTIQ 50	l-DOPA 100	18635 ± 4868**^+^
Effect of 1BnTIQ		*F*_(2/30)_ = 6.18 *P* < 0.01
Effect of l-DOPA		*F*_(2/30)_ = 69.78 *P* < 0.01
Interaction of 1BnTIQ + l-DOPA		*F*_(2/30)_ = 6.19 *P* < 0.01

1BnTIQ was administered acute or chronic during 14 consecutive days at two concentrations (25 and 50 mg/kg i.p.). In the mixed group, l-DOPA (100 mg/kg i.p.) was given once, 15 min after 1BnTIQ administration. Rats were decapitated 2 h after the injections. The results are expressed as the mean ± SEM (*n* = 5–6 animals per group). Data were analyzed with a two-way ANOVA followed by Duncan’s post hoc test. Statistical significance: * P < 0.05, ** *P* < 0.01 versus saline group; ^+^ *P* < 0.05, ^++^ *P* < 0.01 versus l-DOPA group

#### The Effect of Chronic Administration of 1BnTIQ on the l-DOPA-Induced Elevation of Caspase-3 Activity in Rat Hippocampus

**Fig. 3:**
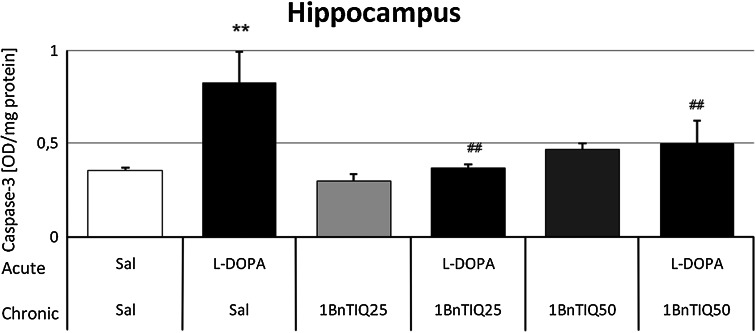
The effect of 1BnTIQ on l-DOPA-induced increase in caspase-3 activity in rat hippocampus. 1BnTIQ was administered chronically at concentrations of 25 or 50 mg/kg i.p. for 14 consecutive days. In the mixed group, l-DOPA (100 mg/kg i.p.) was given once, 30 min after last 1BnTIQ administration. Rats were decapitated 3 h after the last injection. The results are expressed as the mean ± SEM (*n* = 6 animals per group). Data were analyzed with a two-way ANOVA, followed by Duncan’s post hoc test. Statistical significance: **P < 0.01 versus saline-treated group; ^##^ *P* < 0.01 versus l-DOPA-treated group

Chronic systemic administration of 1BnTIQ at a concentration of 25 mg/kg did not alter caspase-3 activity in the hippocampus, whereas a higher concentration of 1BnTIQ (50 mg/kg) produced only a slight increase in this activity (Fig. 3). As expected, acute administration of l-DOPA (100 mg/kg i.p.) induced a significant increase (approx. 100 %; *P* < 0.01) in the activity of caspase-3 in the hippocampus. This effect was completely inhibited by multiple injections of 1BnTIQ at both concentrations (*P* < 0.01) (Fig. 3).

### *In Vivo* Microdialysis

#### The Effects of a Single Administration of 1BnTIQ on l-DOPA-Induced Changes in DA Release in Rat Striatum

**Fig. 4:**
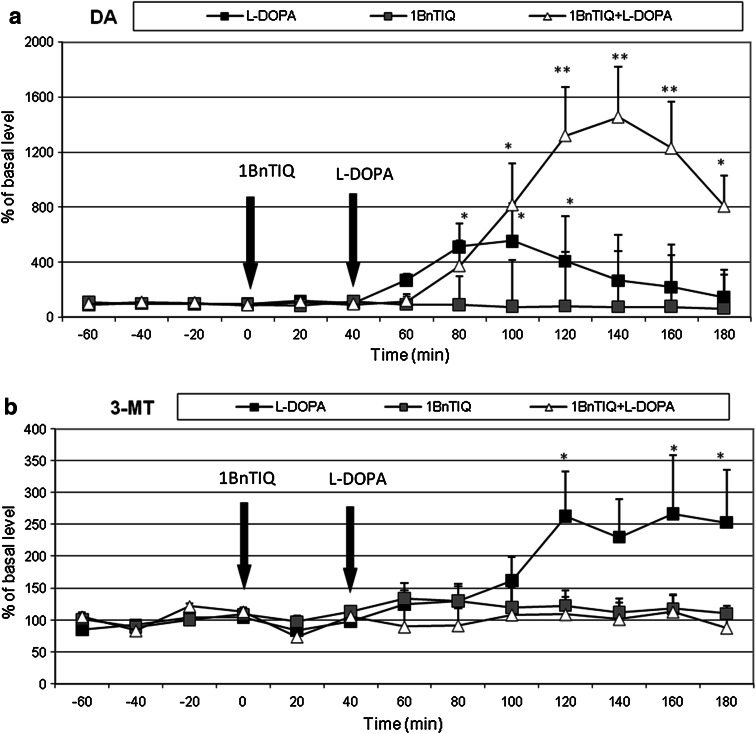
The effects of acute administration of 1BnTIQ on l-DOPA-induced changes in DA release (**a**) and 3-MT concentration (**b**) in rat striatum. Control samples were collected from “–60” to “0;” then, 1BnTIQ (50 mg/kg; at timepoint “0”) or l-DOPA (100 mg/kg; at timepoint “40”) was administered i.p. Dialysates were collected every 20 min. In the mixed group, 1BnTIQ was injected 40 min before l-DOPA administration. The concentration of DA (**a**) and its extraneuronal metabolite 3-MT (**b**) was measured. The basal level of DA in striatum was 10.6 ± 3.1 pg/20 μl. The data are expressed as the mean ± SEM (*n* = 5–6). Statistical significance: *P < 0.05, **P < 0.01 from the basal value (Duncan’s test)

A repeated one-way ANOVA revealed no effect of treatment on DA release into the extracellular space after acute administration of either 1BnTIQ (50 mg/kg i.p.) or l-DOPA (100 mg/kg i.p.) (*F*[2,12] = 3.03, N.S.). However, an effect of time (*F*[12,144] = 4.14, *P* < 0.01) and an interaction between time and treatment were detected (*F*[24,144] = 2.99, *P* < 0.01). Duncan’s post hoc analysis showed that acute l-DOPA administration produced a significant (*P* < 0.01) and long-lasting increase in DA release in the rat striatum (by approximately 500 %) (Fig. 4a). 1BnTIQ alone did not produce a change in DA release. The post hoc test indicated that 1BnTIQ combined with l-DOPA potentiated the effect of l-DOPA and produced a long-lasting increase in the release of DA (up to 1,300 %) (*P* < 0.01) in the striatum (Fig. 4a).

A repeated one-way ANOVA did not reveal any effect of treatment on the concentration of 3-MT after a single injection of 1BnTIQ (50 mg/kg i.p.) or l-DOPA (100 mg/kg i.p.) (*F*[2,12] = 1.15, N.S.). As for DA, an effect of time (*F*[12,144] = 2.09, *P* < 0.05) was detected, but the interaction between time and treatment was not significant (*F*[24,144] = 0.98, N.S.). Duncan’s test indicated that acute l-DOPA administration produced a significant (*P* < 0.05) increase in the concentration of 3-MT in the rat striatum (up to 250 %) (Fig. 4b).

#### The Effects of Multiple Administrations of 1BnTIQ on l-DOPA-Induced Changes in DA Release in Rat Striatum

**Fig. 5:**
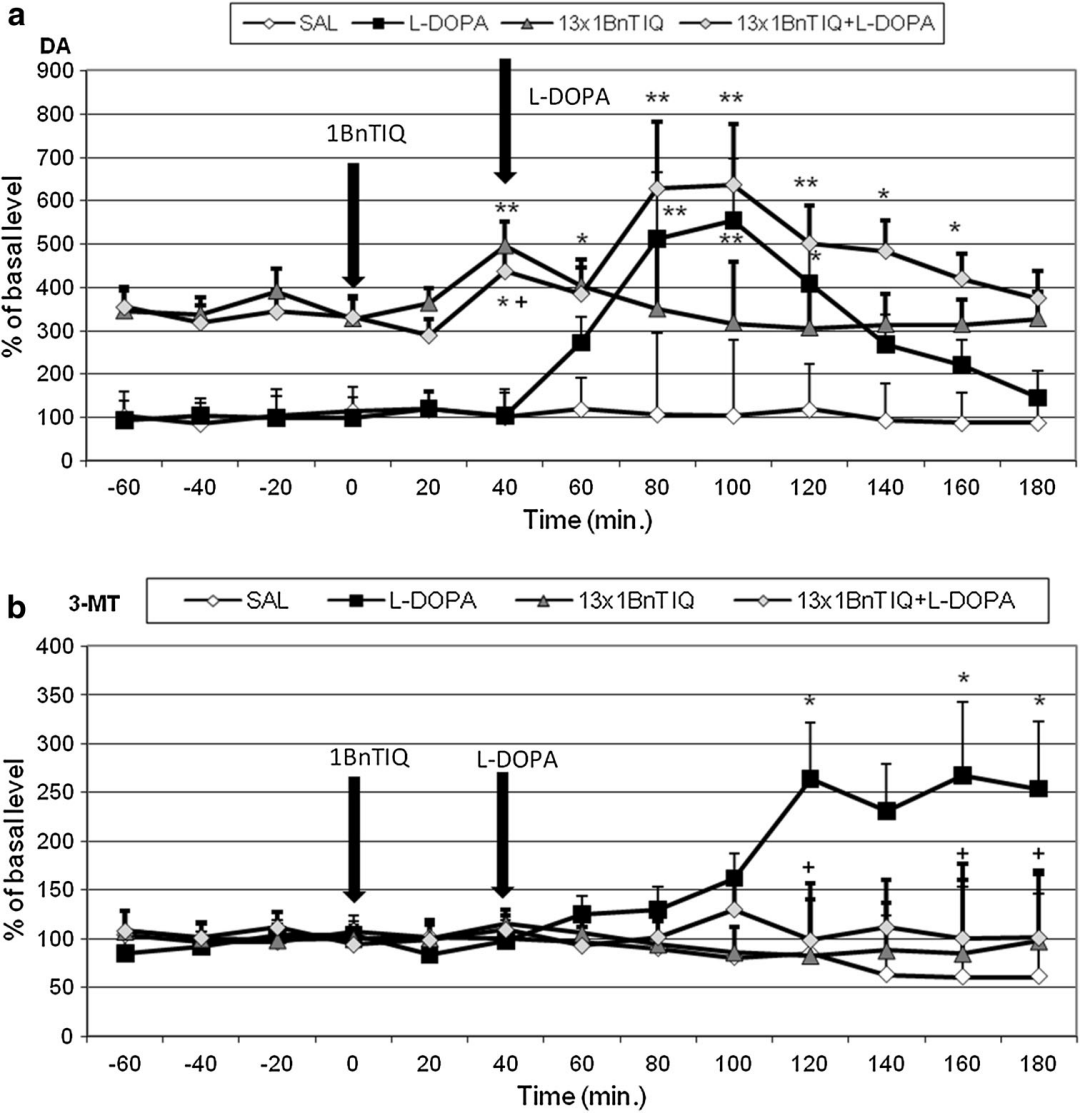
The effects of and chronic administration of 1BnTIQ on l-DOPA-induced changes in DA release (**a**) and 3-MT concentration (**b**) in rat striatum. 1BnTIQ was administered chronic at dose 50 mg/kg i.p. during 14 consecutive days. In the mixed group, l-DOPA (100 mg/kg i.p.) was given once, 40 min after last 1BnTIQ administration. The control group was treated with saline. The dialysate was collected every 20 min. The concentration of DA (**a**) and its extraneuronal metabolite 3-MT (**b**) was measured. The data are expressed as the mean ± SEM (*n* = 5–6). Statistical significance: *P < 0.05, **P < 0.01 from the basal value; ^+^ *P* < 0.05 versus l-DOPA—treated group (Duncan’s test)

A repeated two-way ANOVA showed a significant effect of chronic administration of 1BnTIQ (50 mg/kg i.p.) (*F*[1,18] = 15.56, *P* < 0.01) on DA release (treatment 1), but the effect of acute dose of l-DOPA (treatment 2) was not significant (100 mg/kg i.p.) (*F*[1,18] = 3.1, N.S.). No interaction between treatment 1 and treatment 2 was found (*F*[1,18] = 0.27, N.S.) (Fig. 4a). The statistical analysis demonstrated a significant effect of time (*F*[12,216] = 4.36, *P* < 0.01), as well as an interaction between time and treatment 2 (*F*[12,216] = 5.24, *P* < 0.01). However, there was no interaction between time and treatment 1 (*F*[12,216] = 0.81, N.S.) or between time, treatment 1, and treatment 2 (*F*[12,216] = 0.23, N.S.). Duncan’s post hoc test indicated that acute l-DOPA administration produced a significant (*P* < 0.01) and long-lasting increase in DA release in the striatum (up to 500 %) (Fig. 5a).

A repeated two-way ANOVA showed that chronic administration of 1BnTIQ increased the release of DA in the rat striatum by up to 300 % and, after the last dose, release of DA was elevated by an additional 100 % (Fig. 5a). In the mixed group, chronic administration of 1BnTIQ weakened the effects of l-DOPA when compared to the group treated with l-DOPA alone (Fig. 5a).

A repeated two-way ANOVA found no significant effect of treatment 1 after chronic administration of 1BnTIQ (50 mg/kg i.p.) (*F*[1,18] = 0.66, N.S.) or of treatment 2 after an acute dose of l-DOPA (100 mg/kg i.p.) on the concentration of 3-MT (*F*[1,18] = 2.21, N.S.). Furthermore, there was no interaction between treatment 1 and treatment 2 (*F*[1,18] = 1.41, N.S.) (Fig. 5b). The statistical analysis showed no significant effect of time (*F*[12,216] = 0.57, N.S.), time versus treatment 1 (*F*[12,216] = 0.96, N.S.), or time versus treatment 1 versus treatment 2 (*F*[12,216] = 1.42, N.S.). However, there was a significant interaction between time and treatment 2 (*F*[12,216] = 1.98, *P* < 0.05). Duncan’s post hoc analysis showed that acute l-DOPA administration produced a significant (*P* < 0.05) elevation in the concentration of 3-MT (up to 250 %); this effect was completely antagonized by chronic administration of 1BnTIQ (Fig. 5b).

## Discussion

The main finding of this study was that both acute and chronic systemic administrations of the endogenous neurotoxin 1BnTIQ disturbed the behavioral and biochemical effects of l-DOPA in the rat. Biochemical* ex vivo* studies have shown that administration of l-DOPA (100 mg/kg i.p.), as a precursor of DA, causes a significant increase in DA metabolism, its concentration, and the concentration of all DA metabolites in the extrapyramidal brain structures substantia nigra and striatum. 1BnTIQ, at both investigated concentrations (25 and 50 mg/kg), completely antagonized the effect of l-DOPA in the rat brain. Comparing the results of our behavioral test and our* ex vivo* and* in vivo* biochemical experiments, we observed that 1BnTIQ does not always act in the same direction. In the behavioral and* ex vivo* experiments, 1BnTIQ completely antagonized the l-DOPA-induced biochemical effects, while the* in vivo* microdialysis studies demonstrated the potentiation by 1BnTIQ of l-DOPA-evoked DA release in the striatum (specifically after acute administration of 1BnTIQ; Fig. 4a). Additionally, we have found that chronic administration of 1BnTIQ completely blocks the l-DOPA-induced increase in caspase-3 activity in the hippocampus.

DA was discovered as a neurotransmitter in the animal brain based on its specific regional distribution. 1BnTIQ, like reserpine, a specific inhibitor of vesicular monoamine uptake transporter (VMAT2), produces depletion of striatal DA, such that even synthesized DA is not stored in vesicles efficiently (Wąsik et al. 2009). Reserpine is a model substance frequently used to produce parkinsonism in animals (Colpaert 1987; Lorenc-Koci et al. 1995). Dysfunction of DA neurons has been explicitly correlated with damage to basic transport and storage mechanisms of neurotransmitters [neuronal (DAT) and vesicular (VMAT2) uptake]. The above findings suggest that 1BnTIQ may damage the VMAT2 in dopaminergic neurons, leading to the pathological release of DA into the cytosol. l-DOPA therapy supplements DA deficiencies and increases its metabolism in the brain. Peripheral co-administration of aromatic l-amino acid decarboxylase (AADC) inhibitors (carbidopa, benserazid) improves the bioavailability of l-DOPA (Bartholini et al. 1967). Exogenously administered l-DOPA may be taken up into DA terminals in the brain by the DA reuptake transporter DAT and may be decarboxylated to DA, primarily within AADC-containing cells in the striatum. Therefore, the undisturbed function of DAT is a crucial factor for increasing DA metabolism and the therapeutic effectiveness of l-DOPA in the clinic. Our previous studies, carried out in rat striatum slices, demonstrated that 1BnTIQ significantly inhibits (IC50 in low micromolar concentrations) the DA reuptake transporter (Patsenka et al. 2004). A similar effect was shown by Okada et al. (1998), where 1BnTIQ inhibited the uptake of [^3^H]dopamine through the DAT expressed in HEK293 cells. In light of these findings, DAT is a likely candidate responsible for the selective transport of 1BnTIQ into dopaminergic neurons, leading to the 1BnTIQ neurotoxicity correlated with impairment of DA storage by inhibition of VMAT2 (Wąsik et al. 2009).

**Fig. 6:**
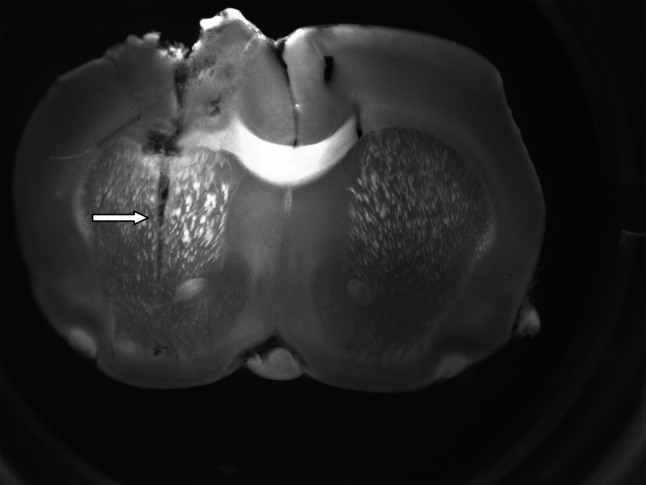
The implantation of the cannula in the rat striatum

Our biochemical* ex vivo* studies showed that administration of l-DOPA (100 mg/kg i.p.) causes an increase in DA and all its metabolites (DOPAC, 3-MT, and HVA) both in the substantia nigra and in the striatum (Table 1). 1BnTIQ at both concentrations (25 and 50 mg/kg i.p.) decreased DA in the nigrostriatal structures (Table 1). In the mixed groups, l-DOPA-induced effects were completely inhibited by both single and multiple administrations of 1BnTIQ. The differences between acute and chronic administration that we observed suggest that, during chronic administration of 1BnTIQ, some tolerance to its DA-depressing effect develops, while the impairment of DA synthesis continues. On the other hand, weakening in the inhibitory action 1BnTIQ after repeated administration may indicate adaptation in the brain and/or brain plasticity. Our* ex vivo* studies suggest that 1BnTIQ prevents the uptake of l-DOPA into the neuron and/or blocks the conversion of l-DOPA into DA. As mentioned, 1BnTIQ antagonism of l-DOPA-evoked increase in DA metabolism could be linked to DAT inhibition by 1BnTIQ (Okada et al. 1998; Patsenka et al. 2004). In fact, the* in vivo* microdialysis results discussed in this paper support this hypothesis. Our results show that 1BnTIQ alone (50 mg/kg i.p.) does not produce significant changes in DA release (Fig. 4a). However Katagiri et al. (2009) reported that acute 1BnTIQ (40 mg/kg i.p.) injection produced a weak increase in the DA release. The discrepancies between our results may be explained through the different areas of the cannula implantation. While our dialysates were collected from central part of the striatum (Fig. 6); in the case of Katagiri et al. (2009) it was done from part of the ventral striatum.

l-DOPA is a direct substrate for COMT, and its metabolism leads to the production of 3-*O*-methyl-dopa (3-MDOPA) in the brain. Therefore, COMT is responsible both for the metabolic inactivation of extraneuronal DA to 3-MT and for the metabolism of l-DOPA to 3-MDOPA (Kopin 1985; Antkiewicz-Michaluk et al. 2001). Selective COMT inhibitors, such as entacapone, tolcapone, and CGP28014, effectively block the O-methylation of l-DOPA and the formation of 3-MDOPA, thus improving its bioavailability and brain penetration (Kaakkola et al. 1994). COMT inhibitors are used as an adjunctive treatment for PD as they increase central l-DOPA availability (Mannisto and Kaakkola 1999). Our *ex vivo* and* in vivo* studies clearly support the notion that 1BnTIQ inhibits COMT-activity, as it significantly decreased the concentrations of both the l-DOPA and DA metabolites, 3-MDOPA and 3-MT, respectively (Table 3; Figs. 4b, 5b).

1BnTIQ given acutely 40 min before l-DOPA (100 mg/kg i.p.) administration, augmented l-DOPA effect and was produced an additional increase in the extraneuronal DA concentration (up to 1,300 %; *P* < 0.01) in striatum (Fig. 4a). However, chronic administration of 1BnTIQ clearly weakens this increase in the l-DOPA effect, likely as a result of the tolerance development (Fig. 5a). The effect of 1BnTIQ connected with the COMT inhibition presented in the paper and discussed above can explained the potentiation of l-DOPA-produced an increase of the extraneuronal DA concentration in microdialysis study (Figs. 4a, 5a).

It is well known that although l-DOPA is the gold standard for the treatment of PD symptoms. l-DOPA can lead to adverse effects, either as a result of oxidative stress because it has the potential to auto-oxidize to a quinine derivative, generating ROS and depleting striatal GSH (Ogawa et al. 1994; Spencer et al. 1996; Hattoria et al. 2009), or by inducing apoptosis, as evidenced by the increased activity of caspase-3 and DNA damage via a mechanism independent of oxidative stress (Pedrosa and Soares-da-Silva 2002; Tanaka and Ogawa 2005; Emdadul Haque et al. 2003). We confirmed these findings with one of our* ex vivo* experiments: acute l-DOPA injection produced a significant increase in caspase-3 activity, an effect that was completely inhibited by chronic administration of 1BnTIQ at both tested concentrations (Fig. 3). It appears that we are observing artificial “neuroprotective” effects of 1BnTIQ, related to its inhibition of DAT activity, and thus of l-DOPA neuronal uptake.

We know from our previous *in vitro* study that 1BnTIQ, at micromolar concentrations, has neurotoxic activity in rat hippocampal cultures, in which it increases apoptotic markers (caspase-3 activity and LDH release; Wąsik et al. 2014). However, follow-up* ex vivo* studies suggested that chronic administration of 1BnTIQ did not have neurotoxic effects on dopaminergic neurons in the substantia nigra, as assessed by tyrosine hydroxylase activity and the level of alpha-synuclein (Wąsik et al. 2014). In contrast, *in vitro* experiments carried out in human dopaminergic cells showed that 1BnTIQ increased alpha-synuclein expression and caused nuclear damage (Shavali et al. 2004).

The results obtained from our behavioral studies are in agreement with the results of the biochemical* ex vivo* analysis in demonstrating both acute and chronic 1BnTIQ antagonisms of l-DOPA-induced locomotor hyperactivity in rat (Figs. 1a, b, 2a, b). However, in the combined groups, stereotyped behaviors linked to l-DOPA administrations, such as sniffing and sometimes licking were still present.

In conclusion, our results demonstrate that 1BnTIQ is not only an endogenous neurotoxin that disrupts the activity of dopaminergic neurons but is also an antagonist of l-DOPA-evoked behavioral and biochemical effects. The data from our* ex vivo* biochemical analysis and* in vivo* microdialysis study suggest that the molecular mechanism of action of 1BnTIQ may involve its inhibition of either or both DAT and COMT activities in the brain. Furthermore, elevated endogenous levels of 1BnTIQ may cause serious adverse effects in PD patients undergoing of l-DOPA therapy.
